# KAN-HyperMP: An Enhanced Fault Diagnosis Model for Rolling Bearings in Noisy Environments

**DOI:** 10.3390/s24196448

**Published:** 2024-10-05

**Authors:** Jun Wang, Zhilin Dong, Shuang Zhang

**Affiliations:** 1 Department of Ocean Engineering, Yantai Institute of Science and Technology, Yantai 265600, China; wangjun201033@163.com; 2 School of Engineering, Zhejiang Normal University, Jinhua 321004, China; 3 School of Computer Science and Technology, Anhui University, Hefei 230601, China; zshuang1031@163.com

**Keywords:** fault diagnosis, hypergraph, Kolmogorov–Arnold Network, KAN-HyperMP

## Abstract

Rolling bearings often produce non-stationary signals that are easily obscured by noise, particularly in high-noise environments, making fault detection a challenging task. To address this challenge, a novel fault diagnosis approach based on the Kolmogorov–Arnold Network-based Hypergraph Message Passing (KAN-HyperMP) model is proposed. The KAN-HyperMP model is composed of three key components: a neighbor feature aggregation block, a feature fusion block, and a KANLinear block. Firstly, the neighbor feature aggregation block leverages hypergraph theory to integrate information from more distant neighbors, aiding in the reduction of noise impact, even when nearby neighbors are severely affected. Subsequently, the feature fusion block combines the features of these higher-order neighbors with the target node’s own features, enabling the model to capture the complete structure of the hypergraph. Finally, the smoothness properties of B-spline functions within the Kolmogorov–Arnold Network (KAN) are employed to extract critical diagnostic features from noisy signals. The proposed model is trained and evaluated on the Southeast University (SEU) and Jiangnan University (JNU) Datasets, achieving accuracy rates of 99.70% and 99.10%, respectively, demonstrating its effectiveness in fault diagnosis under both noise-free and noisy conditions.

## 1. Introduction

In the modern industrial sector, the widespread adoption of “smart manufacturing” and advancements in high-end manufacturing technologies have underscored the importance of enhancing mechanical equipment health management to achieve system intelligence. Rolling bearings, which are essential components of many transmission systems, typically operate under high loads and speeds. Any malfunctions can drastically reduce the efficiency of mechanical devices, potentially leading to significant economic losses and safety incidents. Therefore, developing efficient bearing fault diagnosis technologies is crucial, not only for reducing economic costs, but also for preventing accidents [1].

With the rapid advancement of artificial intelligence technologies, data-driven fault diagnosis has emerged as a research hotspot, focusing primarily on machine learning and deep learning methods [2]. Traditional mechanical fault signal processing techniques include analyses in the time domain, frequency domain, and time-frequency domain. These methods are typically integrated with machine learning technologies such as multilayer perceptrons (MLPs), support vector machines (SVMs), and Bayesian estimation, and are well-suited for diagnosing data with distinct features and straightforward patterns. In contrast, deep learning, an advanced algorithmic approach, offers robust capabilities for automatic feature extraction. It can process large volumes of data and reduce reliance on expert knowledge, significantly improving the efficiency and accuracy of fault diagnosis. Notable deep learning techniques include convolutional neural networks (CNNs) [3,4,5,6], autoencoders (AEs) [7], generative adversarial networks (GANs) [8], and adversarial deep learning (ADL) [9]. The adoption of these technologies not only introduces a new perspective on mechanical fault diagnosis but also fosters the advancement of the entire industrial system towards greater efficiency and safety.

Graph theory models exhibit unique advantages in comprehensively describing fault characteristic information. To effectively handle graph data, Graph Neural Networks (GNNs) have emerged as a burgeoning field. Specifically designed for graph signal processing, GNNs enable the precise definition of values and connections between nodes, capturing and analyzing information from a global perspective. Recently, GNN technology has been applied to fault diagnosis by researchers, in order to deepen their understanding and address fault issues more effectively. GNNs enhance data extraction and inference by aggregating information from neighbors at various depths. These networks have been successfully applied in multiple domains, including physical models [10], chemical structures [11], social networks [12], natural language processing [13], and image classification [14]. For example, Li et al. [15] utilized GNNs to model and analyze graph data, proposing three graph construction methods, exploring seven types of graph convolution networks (GCNs), and four different graph pooling methods. They further developed an intelligent fault diagnosis and predictive diagnosis framework based on GNNs. Additionally, Zhao et al. [16] introduced a semi-supervised graph convolutional deep belief network and applied it to electromechanical system fault diagnosis, which achieved significant diagnostic results, even with limited labeled samples. These studies, which converted vibration signals into graph data and utilized GNNs for fault diagnosis, demonstrate the feasibility and advantages of GNNs in this field.

Graph-based models are becoming a prominent trend in rolling bearing fault diagnosis because they effectively capture the relationships between sample data. However, traditional graph models are limited by their focus on learning pairwise correlations between adjacent samples, as each edge connects only two nodes, making them inadequate for capturing the more complex higher-order relationships that are crucial in practical applications [17]. For instance, during the monitoring of bearing degradation, consecutive samples are not only interrelated but also collectively reflect the component’s gradual deterioration. To illustrate the intricate relationships among multiple samples in fault diagnosis, some researchers have turned to hypergraph structures to represent equipment monitoring data. Hypergraphs connect multiple nodes through hyperedges, enabling a more comprehensive depiction of complex relationships among multisample data. Consequently, hypergraphs are used to represent intricate higher-order relationships between vertices and model complex networks and systems with high-order interactions. Zhang et al. [18] transformed motion current signals into a hypergraph structure and developed a Hypergraph GCN (HGCN) to learn the higher-order relationships between nodes for fault classification. Similarly, Shi et al. [19] transformed vibration signal samples into a hypergraph and mined the high-order structural information between samples using HGCN layers. Yan et al. [20] structured the sample data into multiple hypergraph structures to better learn the high-order data hidden among the samples. Additionally, Feng et al. [21] introduced the Hypergraph Neural Network (HGNN), a model that naturally extends the spectral method of GCN to hypergraphs, and designed corresponding hypergraph convolution operations. Meanwhile, Yadati et al. [22] developed the HyperGCN model, addressing semi-supervised classification problems on hypergraphs. These advancements have promoted the application of hypergraph models in fields such as computer vision [23,24], recommendation systems [25,26], and spatiotemporal forecasting [27,28], achieving significant success. Notably, in the analysis of bearing monitoring data, utilizing hypergraph methods to explore high-order relationships between samples offers a new perspective and methodology for rolling bearing fault diagnosis.

To effectively capture higher-order relationships, Wang et al. [29] introduced T-spectral convolution, a technique specifically designed for handling complex data structures, with a particular strength in representing hypergraphs as tensors. By leveraging the multidimensional characteristics of tensors, this method effectively captures complex inter-node relationships, thereby enhancing the understanding and management of patterns within multidimensional datasets. T-spectral convolution not only captures higher-order relationships but also clearly articulates the multidimensional relationships of data through its intuitive tensor representation, making the intrinsic structure and connectivity more apparent. Additionally, it offers significant flexibility for analyzing complex systems involving various types of interactions. However, T-spectral convolution faces several challenges in practical applications. Constructing and computing large tensors demands substantial computational resources, especially when dealing with large-scale data, potentially leading to reduced processing efficiency. Moreover, as data scales increase, the scalability of T-spectral convolution may become limited, restricting its potential applications on large-scale datasets.

To address the limitations of T-spectral convolution in handling higher-order relationships, the innovative KAN-HyperMP model is introduced in this paper. KAN-HyperMP utilizes $M^{\mathrm{th}}$-order hyperedges within the hypergraph to capture interactions between target nodes and their neighbors, thereby enhancing the model’s learning capabilities and prediction accuracy. The model has been validated on two rolling bearing datasets, demonstrating high fault detection precision even under strong noise interference.

1. An innovative algorithmic framework, KAN-HyperMP, is introduced, specifically designed to manage complex graph structures and high-order data interactions, proving highly effective in applications such as graph-based rolling bearing fault diagnosis;
2. A neighbor feature aggregation block is designed to utilize hypergraph structures, enabling the effective management of complex node interactions by defining and capturing high-order relationships within the hypergraph;
3. A feature fusion block is introduced, integrating node-specific features with those of their neighbors to provide a comprehensive view of local graph structures, thereby significantly enhancing prediction accuracy;
4. A KANLinear block, based on the Kolmogorov–Arnold theorem and employing B-spline functions as activation functions, is introduced to effectively suppress noise, enhancing the model’s robustness and generalization capabilities in noisy environments.

The rest of this paper is as follows: The proposed model is introduced in Section 2. In Section 3, experiments are carried out, and the effectiveness of the proposed method is analyzed. The Section 4 summarizes and puts forward the avenues for future work.

## 2. Proposed Model

In the task of rolling bearing fault diagnosis, fault samples are unstructured, making it challenging to construct a hypergraph that can represent the hidden structure within sample data and across different samples. To address this issue, a hypergraph construction method capable of capturing the data structure among fault samples is proposed, and a corresponding neural network is developed based on the constructed hypergraph for fault identification.

### 2.1. Hypergraph Construction

Compared to traditional graph structures, hypergraphs are unique in their ability to connect multiple nodes through hyperedges, facilitating the modeling of higher-order relationships. A hypergraph G=(V,E) is defined, where $V=\{v_{1},v_{2},\ldots ,v_{N}\}$ represents a set of *N* nodes (or vertices), and $E=\{e_{1},e_{2},\ldots ,e_{K}\}$ comprises *K* hyperedges. Each hyperedge $e_{k}$ can be defined as follows:

(1) $$e_{k}=\left\{v_{i}|v_{i}\in V\;\mathrm{and}\;v_{i}\;\mathrm{is}\;\mathrm{part}\;\mathrm{of}\;\mathrm{hyperedge}\;e_{k}\right\},\;k=1,2,\ldots ,K$$

In a hypergraph G, the maximum edge cardinality m.c.e(G) indicates the maximum number of nodes contained in any hyperedge, mathematically defined as *M*:

(2) $$M=\max_{e_{k}\in E}\left|e_{k}\right|$$

Hypergraphs depict the connectivity between nodes through an incidence matrix $H\in R^{\left|V|\times |E\right|}$. In this matrix, each element H(v,e) is defined as follows:

(3) $$H\left(v,e\right)=\left\{\begin{array}{cc} 1, & \mathrm{if}\;v\in e\\ 0, & \mathrm{if}\;v\notin e \end{array}\right.$$

This implies that when a node *v* in the hypergraph is associated with a hyperedge *e*, the corresponding element in the matrix is 1; otherwise, it is 0.

The above metrics reflect the fundamental structural features of the hypergraph, crucial for the analysis and processing of datasets based on hypergraphs. The quality of hypergraph construction significantly impacts model training and the accuracy of fault diagnosis, as all HGNN utilize the hypergraph, specifically the incidence matrix *H*, to capture information between nodes (samples). Therefore, constructing a hypergraph is a critical step in using HGNN for fault diagnosis tasks. However, commonly used datasets in fault diagnosis, such as SEU, JNU, CWRU, etc., do not provide explicit hypergraph structural information, as there are no clear connections among the samples in these datasets. Consequently, it becomes necessary to manually design a hypergraph structure that can accurately reflect the relationships between different sample signals within these datasets.

From the initial vibration signals, $X=\{X_{1},X_{2},X_{3},\ldots ,X_{n}\}$, we resample each sample signal feature using a predefined set of sampling frequencies $R=\{r_{1},r_{2},r_{3},\ldots ,r_{m}\}$. Here, $r_{1}$ serves as the base sampling frequency, with subsequent frequencies defined as $r_{2}=\frac{1}{2}r_{1}$, $r_{3}=\frac{1}{4}r_{1}$, and so on, until $r_{m}=\frac{1}{2^{m-1}}r_{1}$. The resampled results for all original samples are generated by this method:

(4) $$\left\{\begin{array}{c} X^{r_{1}}=\left\{X_{1}^{r_{1}},X_{2}^{r_{1}},\ldots ,X_{n}^{r_{1}}\right\}\\ X^{r_{2}}=\left\{X_{1}^{r_{2}},X_{2}^{r_{2}},\ldots ,X_{n}^{r_{2}}\right\}\\ \vdots \\ X^{r_{m}}=\left\{X_{1}^{r_{m}},X_{2}^{r_{m}},\ldots ,X_{n}^{r_{m}}\right\} \end{array}\right.$$

To more precisely capture signal characteristics at different time points, we apply sliding window resampling to the signals $X^{r_{1}},X^{r_{2}},\ldots ,X^{r_{m}}$. This approach enables the extraction of local features from the continuous signals, providing the model with coherent and comprehensive temporal feature data.

The newly acquired signal feature data then undergo Min–Max Normalization to ensure the numerical stability of the model calculations and to mitigate errors due to large or small numerical ranges. After normalization, a Fast Fourier Transform (FFT) is performed to convert the signals into the frequency domain. The processed results are as follows:

(5) $$\left\{\begin{array}{c} X_{norm,f}^{r_{1}}=\left\{X_{1,norm,f}^{r_{1}},X_{2,norm,f}^{r_{1}},\ldots ,X_{n,norm,f}^{r_{1}}\right\}\\ X_{norm,f}^{r_{2}}=\left\{X_{1,norm,f}^{r_{2}},X_{2,norm,f}^{r_{2}},\ldots ,X_{n,norm,f}^{r_{2}}\right\}\\ \vdots \\ X_{norm,f}^{r_{m}}=\left\{X_{1,norm,f}^{r_{m}},X_{2,norm,f}^{r_{m}},\ldots ,X_{n,norm,f}^{r_{m}}\right\} \end{array}\right.$$

To facilitate the model’s ability to capture inherent connections between samples, features from samples with identical resampling frequencies and the same fault type are concatenated. For instance, if $X_{1}$ and $X_{i}$ are both classified as having an inner ring fault, their features are concatenated to form $X_{1,i}^{r_{1}},X_{1,i}^{r_{2}},\ldots ,X_{1,i}^{r_{m}}$, as shown in Equation (6).

(6) $$\left\{\begin{array}{c} X_{1,i}^{r_{1}}=X_{1,norm,f}^{r_{1}}\|X_{i,norm,f}^{r_{1}}\\ X_{1,i}^{r_{2}}=X_{1,norm,f}^{r_{2}}\|X_{i,norm,f}^{r_{2}}\\ \vdots \\ X_{1,i}^{r_{m}}=X_{1,norm,f}^{r_{m}}\|X_{i,norm,f}^{r_{m}} \end{array}\right.$$

**Figure 1:**
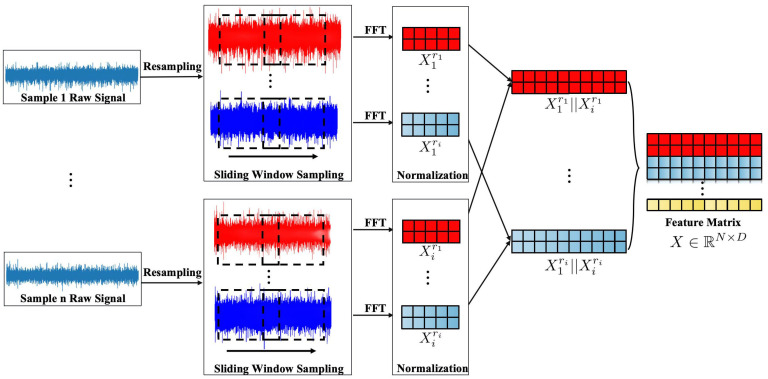
The construction process of Feature Matrix.

Finally, to ensure each sample is accurately classified, the samples obtained through the above process are vertically stacked, forming a feature matrix $X\in R^{N\times D}$. The entire process is illustrated in Figure 1.

**Figure 2:**
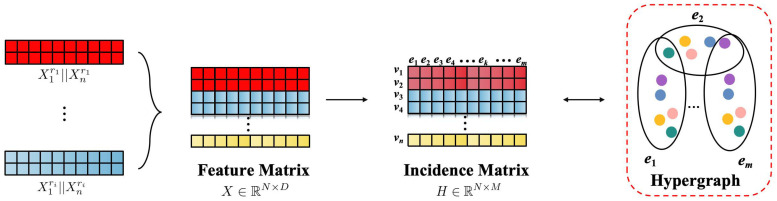
The construction process of hypergraph.

Additionally, to construct the hypergraph, it is crucial to establish connections between nodes and define hyperedges. The K-Nearest Neighbors (KNN) algorithm is used to calculate the Euclidean distances between sample features, forming the incidence matrix $H\in R^{N\times M}$, as shown in Figure 2.

### 2.2. T-Spectral Convoluation

In hypergraphs, a hyperedge that connects multiple nodes can collectively represent higher-order relationships, such as the collective behaviors or attributes of a node group. This multi-node relationship is a core feature of hypergraphs and is crucial for understanding interactions within complex systems. Traditional matrix-based methods, such as incidence matrices, often fail to adequately represent higher-order relationships by reducing the hypergraph’s multiway connections to pairwise interactions, leading to a loss of crucial multiway interaction information originally present in the data.

Building on this analysis, research has introduced the hypergraph T-spectral Convolution [29], which leverages tensor representations and t-product decompositions to enable the direct manipulation of hypergraph data in higher dimensions. This approach allows models to handle higher-order relationships more naturally, overcoming the limitations of traditional methods that reduce high-order hypergraphs to two-dimensional matrices. The t-product, a powerful tool for complex algebraic operations, preserves the multidimensional structure of the data, thereby capturing the deep structures and patterns within the hypergraph. The formula is expressed as follows:

(7) $$Z_{s}=A_{s}^{\mathrm{norm}}*X_{s}*W_{s}$$

Here, $A_{s}^{\mathrm{norm}}$ is the normalized adjacency tensor, and $X_{s}\in R^{N\times D\times N^{\left(M-2\right)}}$ represents the CNI signal tensor, defined as follows:

Given a feature (or signal) matrix $X\in R^{N\times D}$, where *N* is the number of nodes in the hypergraph and *D* is the feature dimension for each node, the interaction of all nodes along the *d*-th dimension (d=1,…,D) is given by

(8) $$\mathrm{CNI}\left({\left[x\right]}_{d}\right)=\underset{(M-1)\;\mathrm{times}\;}{\underset{︸}{{\left[x\right]}_{d}\circ {\left[x\right]}_{d}\ldots \circ {\left[x\right]}_{d}}}\in R^{N\times 1\times N^{\left(M-2\right)}}$$

where ∘ denotes the outer product (also known as the basic tensor product), and ${\left[x\right]}_{d}\in R^{N}$ represents the *d*-th dimensional feature vector of the nodes.

While T-spectra convoluation offers numerous theoretical advantages, such as the ability to process high-order neighbor information, it also faces significant drawbacks, including high computational complexity and substantial memory requirements. For instance, in Equation (7), $X_{s}\in R^{N\times D\times N^{\left(M-2\right)}}$ describes a high-dimensional tensor. While constructing such a tensor is feasible for small hypergraphs, it becomes impractical for larger hypergraphs, such as those in this paper, due to computational limitations.

### 2.3. Proposed Model

**Figure 3:**
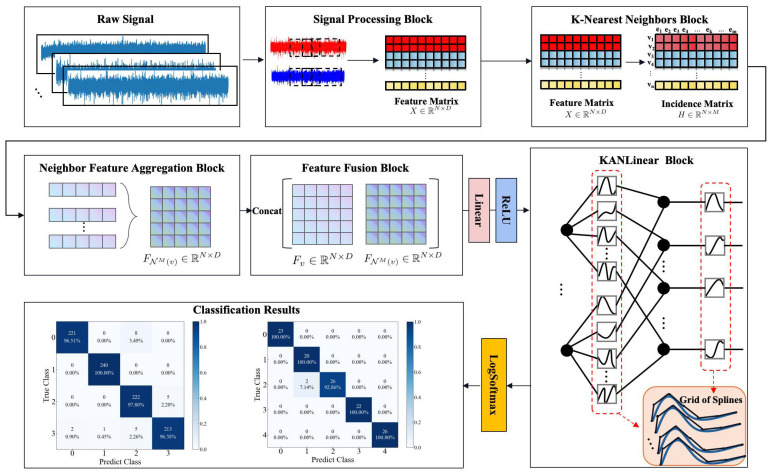
The architecture overview of our KAN-HyperMP. The raw signal is processed into the final signal feature matrix *X* using techniques such as resampling and sliding window sampling. An incidence matrix *H* is then constructed using the KNN algorithm, establishing a hypergraph structure. Based on the hypergraph, the neighbor feature aggregation block extracts information from high-order neighbor nodes. This information is then integrated with the node’s own information through the feature fusion block. Finally, feature extraction is completed using the KANLinear block, facilitating fault diagnosis.

To efficiently capture higher-order neighbor features, similar to hypergraph T-spectral convolution, while minimizing the computational complexity of high-dimensional tensors, a novel model called KAN-HyperMP is introduced in this paper. KAN-HyperMP is mainly divided into three parts: a neighbor feature aggregation block, a feature fusion block, and KANLinear block. The overall model is depicted in Figure 3.

**Figure 4:**
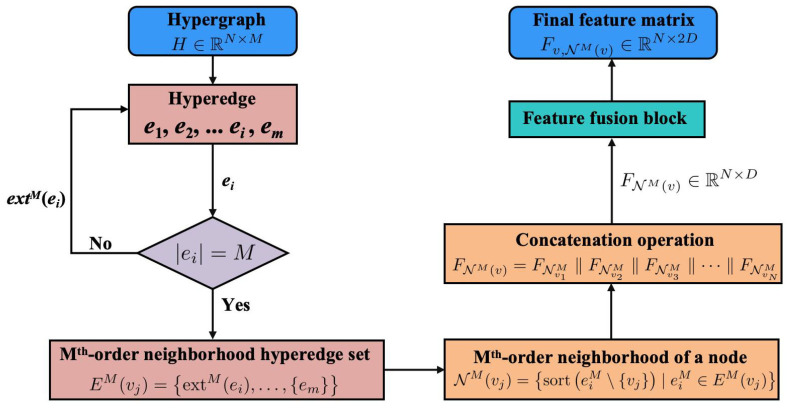
Flowchart of the proposed neighbor feature aggregation block.

Figure 4 illustrates the flowchart for the neighbor feature aggregation block process when provided with a hypergraph structure. This block first checks if the number of nodes in a hyperedge equals *M*. If not, the hyperedge is expanded. Once all hyperedges satisfy this condition, both the $M^{\mathrm{th}}$-order neighborhood hyperedge set and the $M^{\mathrm{th}}$-order neighborhood of a node are calculated. Subsequently, the node’s high-order neighbor features are acquired through a concatenation operation. Finally, the feature fusion block processes these to generate the final feature vector representation, with Section 2.3.1 and Section 2.3.2 providing detailed explanations of the neighbor feature aggregation block and the feature fusion block, respectively.

#### 2.3.1. Neighbor Feature Aggregation Block

The design of the neighbor feature aggregation block is based on hypergraph theory, utilizing high-order neighborhood relationships to expand the adjacency information in traditional graph structures. This method aims to effectively extract and integrate features from adjacent nodes within the hypergraph, thereby capturing the complex interactions and relationships between nodes. By processing more complex data structures and understanding deeper dependencies among nodes, the model’s predictive capabilities and learning efficiency are significantly enhanced by this block.

When the working principles of this module are introduced, two fundamental concepts in hypergraphs are first presented: the $M^{\mathrm{th}}$-order neighborhood hyperedge set and the $M^{\mathrm{th}}$-order neighborhood. These concepts provide a crucial theoretical foundation for understanding how the block processes data.

**Figure 5:**
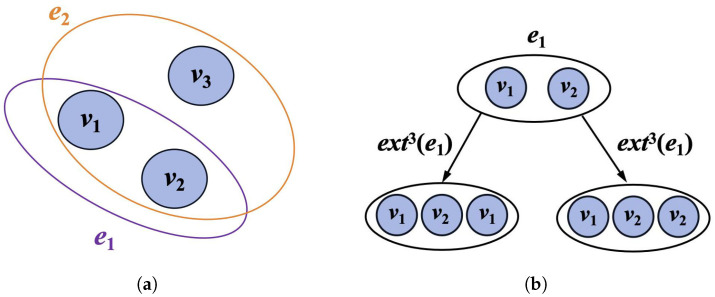
Construct the 3rd-order neighborhood hyperedge set for node $v_{1}$. (**a**) Hypergraph structure. (**b**) Expand hyperedge.

1. **$M^{\mathrm{th}}$-order neighborhood hyperedge set**
  In defining the $M^{\mathrm{th}}$-order hyperedges within a hypergraph G=(V,E), scenarios are differentiated based on the number of nodes each hyperedge contains:
  (9) $$e^{M}=\left\{\begin{array}{cc} \{e\}, & \mathrm{if}\;|e|=M,\\ \left\{ext^{M}\left(e\right)|\left|ext^{M}\left(e\right)\right|=M\right\}, & \mathrm{if}\;|e|<M \end{array}\right.$$
  Based on this, an $M^{\mathrm{th}}$-order neighborhood hyperedge set can be defined for each hyperedge as follows:
  (10) $$E^{M}\left(v\right)=\left\{e^{M}|e\in E,v\in e\right\}$$
2. **$M^{\mathrm{th}}$-order neighborhood of a node**
  Building on Equations (9) and (10), the $M^{\mathrm{th}}$-order neighborhood of a node can be defined as follows:
  (11) $$N^{M}\left(v\right)=\left\{\mathrm{sort}\left(e^{M}\backslash \left\{v\right\}\right)|e^{M}\in E^{M}\left(v\right)\right\}$$
  where $e^{M}\backslash \left\{v\right\}$ denotes the removal of the target node *v* from the set $e^{M}$ and the sort function refers to the ordering of the remaining nodes. This structured definition of neighborhoods offers an effective method for processing and analyzing hypergraph data, significantly enhancing the model’s comprehension of complex node relationships.
  For instance, consider a simple hypergraph as shown in Figure 5a, and based on Equation (2); M=3 is determined. According to the previously defined method, hyperedge $e_{1}$ is initially expanded to obtain ${ext}^{3}\left(e_{1}\right)$, as shown in Figure 5b. Based on the previously defined criteria, the 3rd-order neighborhood hyperedge set for node $v_{1}$ is determined, as shown in Equation (12).
  (12) $$E^{3}\left(v_{1}\right)=\left\{{\mathrm{ext}}^{3}\left(e_{1}\right),\left\{e_{2}\right\}\right\}=\left\{\left\{\left(v_{1},v_{2},v_{1}\right),\left(v_{1},v_{2},v_{2}\right)\right\},\left\{(v_{1},v_{2},v_{3})\right\}\right\}$$
  Subsequently, the final 3rd-order neighborhood for $v_{1}$ can be represented as follows:
  (13) $$N^{3}\left(v_{1}\right)=\left\{\left\{\mathrm{sort}\left(v_{2},v_{1}\right),\mathrm{sort}\left(v_{2},v_{2}\right)\right\},\left\{\mathrm{sort}(v_{2},v_{3})\right\}\right\}$$
  Using this method, neighboring nodes within different hyperedges for other nodes can also be identified. For instance, the 3rd-order neighborhoods for $v_{2}$ and $v_{3}$ are as follows:
  (14) $$N^{3}\left(v_{2}\right)=\left\{\left\{\mathrm{sort}\left(v_{1},v_{1}\right),\mathrm{sort}\left(v_{1},v_{2}\right)\right\},\left\{\mathrm{sort}(v_{1},v_{3})\right\}\right\}$$
  (15) $$N^{3}\left(v_{3}\right)=\left\{\left\{\mathrm{sort}(v_{1},v_{2})\right\}\right\}$$
  This structured approach allows us to easily determine high-order neighbors for each target node within different hyperedges, facilitating the aggregation of features through a specified algorithm to enhance cross-node interactions. After the above concepts have been introduced, a detailed explanation of how the neighbor feature aggregation block performs neighbor feature aggregation will now be provided. The core of the neighbor feature aggregation block is mainly divided into the following two steps:

**Step 1**: High-order neighbors features.

Consider node $v_{1}$, whose 3rd-order neighborhood is defined as

(16) $$N^{3}\left(v_{1}\right)=\left\{\left\{\mathrm{sort}\left(v_{2},v_{1}\right),\mathrm{sort}\left(v_{2},v_{2}\right)\right\},\left\{\mathrm{sort}(v_{2},v_{3})\right\}\right\}$$

The neighborhood features for node $v_{1}$ are

(17) $$F_{N_{v_{1}}^{3}}=P_{1}\cdot \left(x_{v1}⊙x_{v2}\right)+P_{2}\cdot \left(x_{v2}⊙x_{v2}\right)+P_{3}\cdot \left(x_{v2}⊙x_{v3}\right)$$

where $x_{v1}$, $x_{v2}$, and $x_{v3}$ are the feature vectors of nodes $v_{1}$, $v_{2}$, and $v_{3}$ respectively, and $P_{1}$, $P_{2}$, $P_{3}$ correspond to the combinatorial counts from sort(·). The ⊙ operation denotes the Hadamard (element-wise) product along the feature dimension.

**Step 2**: Hyperedge weights.

Notably, hyperedge $e_{1}$ includes two nodes, while $e_{2}$ includes three. To capture the variation among hyperedges during feature aggregation, a weight for each hyperedge ($W_{e}$) is introduced, calculated as follows:

(18) $$W_{e}=\frac{\left|e\right|}{\alpha }$$

where α=∑i=0|e|(−1)i|e|i(|e|−i)M.

Therefore, the final 3rd-order neighborhood feature for node $v_{1}$ is as follows:

(19) $$F_{N_{v_{1}}^{3}}=W_{e_{1}}\cdot P_{1}\cdot \left(x_{v1}⊙x_{v2}\right)+W_{e_{1}}\cdot P_{2}\cdot \left(x_{v2}⊙x_{v2}\right)+W_{e_{2}}\cdot P_{3}\cdot \left(x_{v2}⊙x_{v3}\right)$$

Repeating this process for all target nodes enables us to obtain neighbor features that can be extended to the $M^{\mathrm{th}}$-order, resulting in the final $M^{\mathrm{th}}$-order neighbor features, as defined in Equation (20).

(20) $$F_{N^{M}\left(v\right)}=F_{N_{v_{1}}^{M}}\|F_{N_{v_{2}}^{M}}\|F_{N_{v_{3}}^{M}}\|\cdots \|F_{N_{v_{N}}^{M}}$$

where $F_{N^{M}\left(v\right)}\in R^{N\times D}$|| represents the concatenation operation.

#### 2.3.2. Feature Fusion Block

By integrating node-specific features with those of their neighbors, the model transitions from a “micro” to a “macro” perspective. This shift enhances the understanding of each node’s role and impact within its neighborhood, helping to capture a more comprehensive view of the local graph structure. Additionally, integrating these features facilitates effective fusion through the feature fusion block, defined by the following formula:

(21) $$F_{v,N^{M}\left(v\right)}=\sigma \left[\mathrm{MLP}\left(\mathrm{COMBINE}\left(F_{v},F_{N^{M}\left(v\right)}\right)\right)\right]$$

where $F_{v}\in R^{N\times D}$ represents the node’s own feature vector, and σ denotes the activation function, with ReLU being the choice in this study. The function COMBINE is defined as follows:

(22) $$\mathrm{COMBINE}\left(F_{v},F_{N^{M}\left(v\right)}\right)=\left[\begin{array}{cc} F_{v} & F_{N^{M}\left(v\right)} \end{array}\right]$$

This method involves concatenating features along dimension *D*, preserving all original feature information from the participating nodes and ensuring that both the node’s and its neighbors’ features are clearly represented in the final feature matrix.

#### 2.3.3. Kanlinear Block

Drawing inspiration from the Kolmogorov–Arnold theorem, the literature [30] introduces the KAN, which uniquely applies activation functions. Unlike traditional neural networks that apply activation functions to each node, KANs implement these functions on the edges rather than the nodes themselves. Additionally, KANs leverage B-spline functions as activation functions due to their superior approximation capabilities, which significantly enhance the network’s ability to learn and model complex data relationships. The functional form of the KAN is defined as follows:

(23) $$\phi \left(x\right)=w_{b}b\left(x\right)+w_{s}\;\mathrm{spline}\left(x\right)$$

where b(x) serves as the basis function, given by $b\left(x\right)=\mathrm{silu}\left(x\right)=\frac{x}{1+e^{-x}}$; the spline function spline(x) is parameterized as a linear combination of B-splines:

(24) $$\mathrm{spline}\left(x\right)=\sum_{i}c_{i}B_{i}\left(x\right)$$

**Figure 6:**
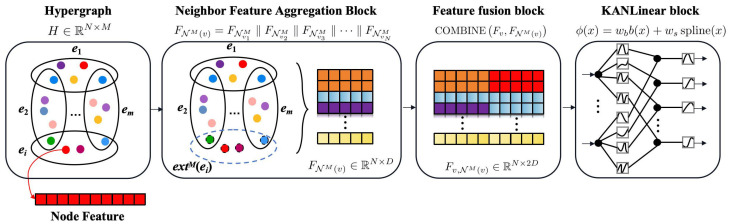
The architecture of KAN-HyperMP.

Owing to the smooth nature of B-spline activation functions, which possess significant noise suppression characteristics, these functions effectively dampen random fluctuations in input data, thereby enhancing the network’s stability and predictive accuracy in noisy environments. In the experimental section, KAN is replaced with a traditional Multilayer Perceptron (HyperMP-MLP), and a comparative analysis is conducted with the KAN’s results, further affirming the method’s effectiveness. The overall architecture of the KAN-HyperMP model is shown in Figure 6.

## 3. Experiments Description

In this section, the effectiveness of the constructed model is validated using two open-source bearing fault diagnosis datasets: SEU and JNU. Experiments are conducted on a server equipped with an Intel(R) Xeon(R) CPU and an NVIDIA L4 GPU. The network framework is implemented in a PyTorch 2.3.1 and CUDA 12.1 environment. KAN-HyperMP has a hidden dimension of 256, a combined neighbor feature aggregation block and feature fusion block, and a single KANLinear block for final feature extraction. For constructing the incidence matrix with the KNN algorithm, the number of nearest neighbors (K) is set to 3, which accordingly sets the model’s M value to 3. The model training employs a negative log-likelihood loss function and is optimized using the Adam algorithm with a learning rate of $1\times 10^{-3}$ and a weight decay rate of $5\times 10^{-6}$. In order to evaluate the model’s performance, the datasets are split into training, validation, and test sets with a ratio of 60%, 20%, and 20%, respectively.

### 3.1. Datasets Description

**Figure 7:**
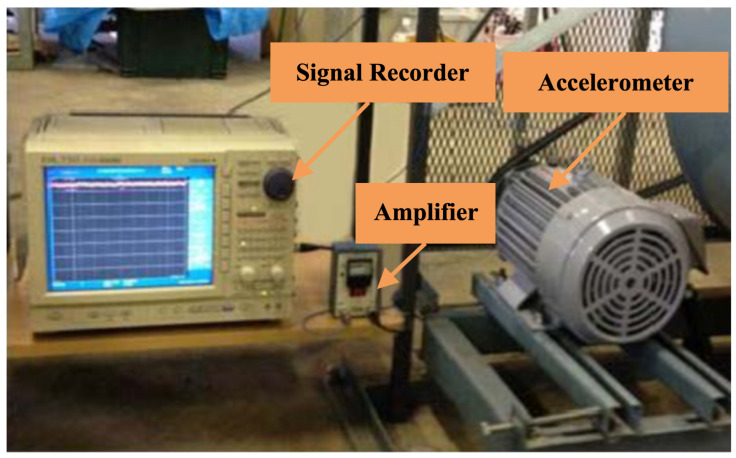
The JNU testbed [31].

Figure 7 shows the JNU testbed, which is composed of a signal recorder, an accelerometer, and an amplifier. The JNU Dataset is primarily used to validate the generalization performance and superiority of the proposed diagnostic model. The bearing vibration signals in this dataset are sampled at a frequency of 50 kHz. The dataset is specifically designed to focus on single fault types, excluding the diagnosis of composite faults. It provides comprehensive documentation of four distinct bearing health states: Normal (N), Inner Race Fault (IB), Outer Race Fault (OB), and Rolling Element Fault (TB), covering a total of four unique fault types.

**Figure 8:**
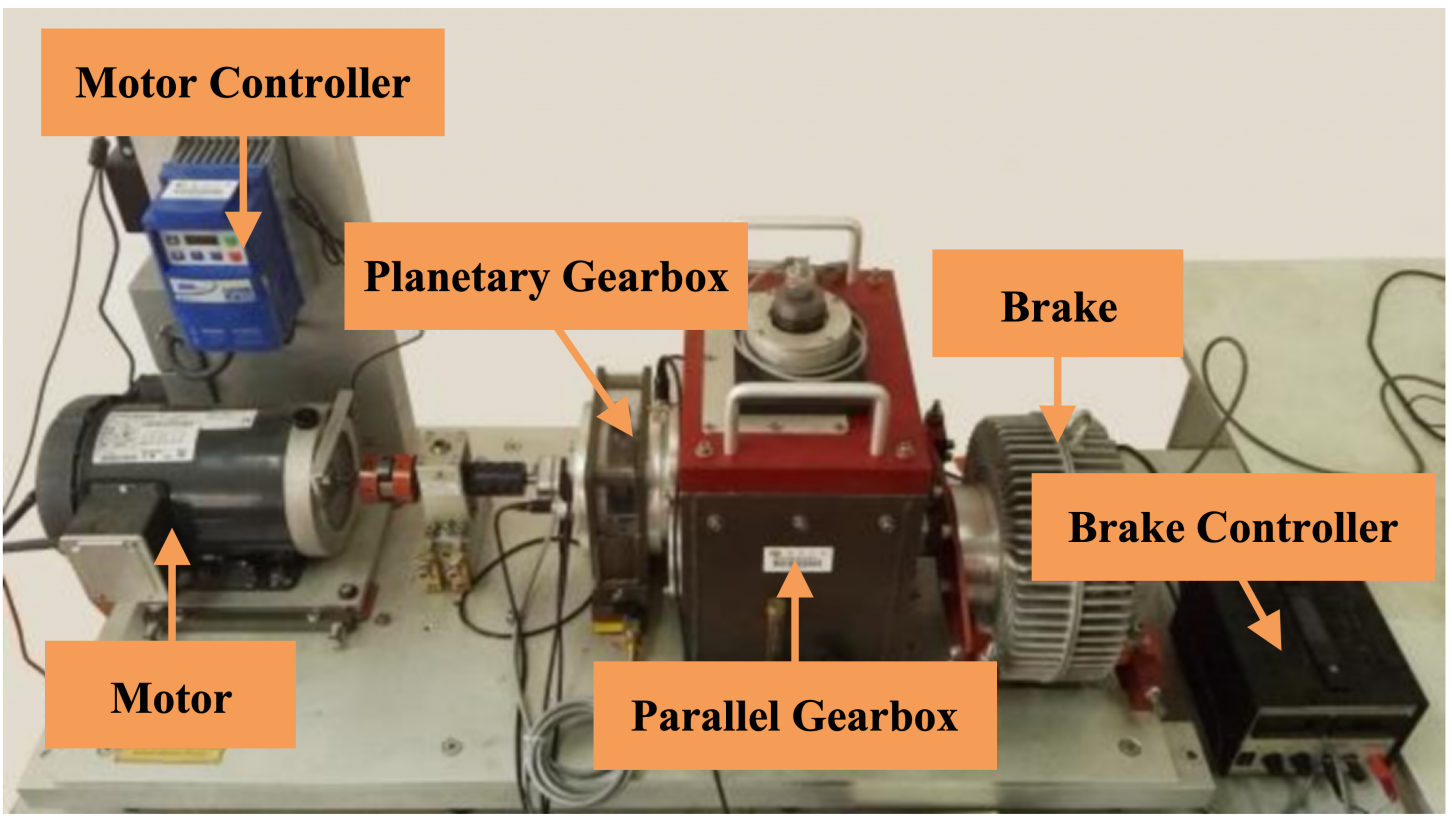
The SEU testbed [31].

The SEU Bearing Dataset, obtained from the Dynamic Drive Simulator (DDS), is tailored specifically for bearing fault diagnosis and learning tasks. The bearing signals in this dataset are sampled at a frequency of 5120 Hz. Data are gathered under two operational settings: 20 Hz-0 V and 30 Hz-2 V, encompassing normal and various faulted conditions. These conditions are categorized into five distinct types: Normal, Ball (defects on the rolling element), Inner (defects on the inner race), Outer (defects on the outer race), and Combination (concurrent defects on both the inner and outer races). This dataset is instrumental for basic bearing fault diagnostics, facilitating transfer learning across different loading conditions and enabling the analysis of complex combined inner and outer race faults. It effectively addresses the diverse requirements of fault diagnostics and predictive maintenance. The SEU testbed is depicted in Figure 8.

### 3.2. Baseline Models

1. GCN [32]: This is a well-established spatial learning model widely used for spatial prediction tasks. This model analyzes vibration data from bearings to detect potential fault patterns;
2. GAT [33]: GAT uses a graph structure and a Graph Attention Network to represent and analyze relationships among bearing monitoring samples, effectively diagnosing faults in rolling bearings;
3. HGNN [21]: This employs a clique expansion to generalize convolutions in hypergraphs, using the hypergraph Laplacian and Chebyshev polynomials to learn complex relationships in bearing data effectively;
4. CNN [34]: This model, consisting of one-dimensional convolutional layers and max-pooling layers, autonomously learns patterns and features from sensor data, including vibration signals, for effective rolling bearing fault diagnosis;
5. LSTM [35]: This model utilizes stacked LSTM units for time series prediction. By analyzing bearing time series data, it effectively identifies fault progression trends;
6. HyperGCN [22]: It is a refined clique expansion method that enhances the hypergraph Laplacian with weighted mediators between vertices. This method boosts fault detection and early diagnosis by enabling efficient complex sensor data analysis.

### 3.3. Experiment Results and Discussion

#### 3.3.1. Demonstration and Analysis without Noise

To minimize the impact of randomness in the experimental results, all models in this study are tested five times under noise-free conditions. Table 1 presents the average accuracy and F1-scores from five repeated experiments across two datasets. These results indicate that the proposed model achieved classification accuracies of 99.70% and 99.10% on the test sets, significantly outperforming all baseline models and confirming the superior fault detection capabilities of our approach.

**Figure 9:**
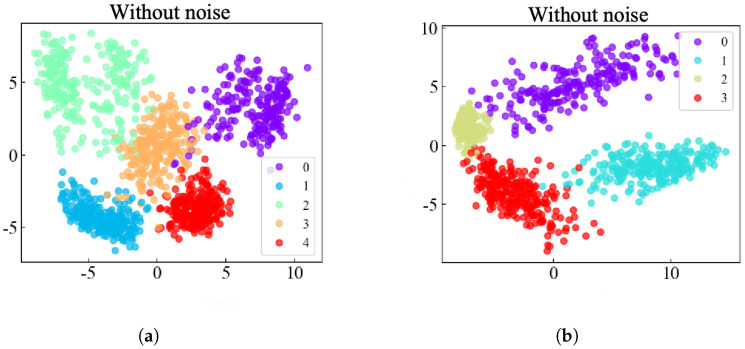
A 2D PCA visualization of rolling bearing fault diagnosis on the SEU and JNU Datasets. (**a**) SEU Dataset. (**b**) JNU Dataset.

To clearly demonstrate the feature extraction prowess of the KAN-HyperMP model, Principal Component Analysis (PCA) is employed to visualize high-dimensional data in two dimensions. This method effectively displays the data embedding vectors learned by the model and the distribution of data across different categories. PCA is a technique used to transform high-dimensional data into a lower-dimensional space for visualization. For this purpose, we used the results from the third experiment for two-dimensional PCA visualization, as illustrated in the Figure 9a,b. The figures show that in the PCA space, the model’s outputs form distinct clusters with significant separation between categories, highlighting the model’s ability to effectively differentiate between various types of samples in a noise-free environment.

#### 3.3.2. Demonstration and Analysis under Strong Noise

**Figure 10:**
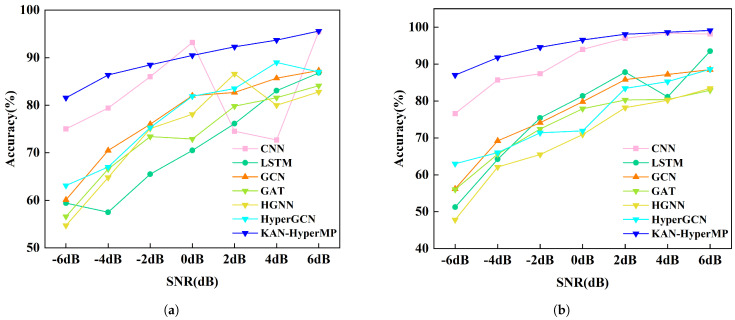
Rolling bearing fault diagnosis accuracies of compared methods at seven noise levels. (**a**) Experimental results on the SEU Dataset. (**b**) Experimental results on the JNU Dataset.

In the operational environment of mechanical equipment, noise generation is inevitable. Consequently, this study incorporates Gaussian white noise at various signal-to-noise ratios (SNR) into the original monitoring data to simulate real-world conditions. The primary purpose of this approach is to assess the model’s noise resistance capabilities. By adding different levels of Gaussian white noise to the original signals, vibration signals under various SNR conditions are generated. To explore the limits of the model’s resistance to noise, we selected a noise level range from −6 dB to 6 dB. Additionally, to eliminate the randomness in the experimental results, each model is tested five times under each SNR scenario. Table 2 and Figure 10a, Table 3 and Figure 10b (showing the average classification results of all models) demonstrate the models’ classification accuracies under extreme noise conditions, ranging from −6 dB to 6 dB.

Notably, at SNR = −6 dB, where noise almost completely masks the original signal features, the accuracy of the HGNN model drops to 54.70% and 47.80% on the SEU and JNU Datasets, respectively, a level considered unsatisfactory. Furthermore, other graph neural network models (such as GCN, GAT and HypergraphGCN) have accuracies between 50% to 60% under SNR = −6 dB conditions, indicating that the models are essentially ineffective in this scenario. This phenomenon may occur because noise in the initial node features introduces anomalous edges into the graph structure or propagates through the network via connections in the adjacency or incidence matrices. Due to the close connections between nodes, incorrect or irrelevant information can quickly spread to multiple nodes, impacting the entire graph’s learning and inference processes.

In contrast, traditional CNNs, with their locally connected features in convolutional layers, can capture local characteristics. Even if part of the input features is affected by noise, other unaffected areas can still effectively provide useful information. Therefore, even under extreme SNR conditions of −6 dB, CNNs maintain approximately 70% accuracy on the SEU and JNU Datasets. LSTMs, designed for processing sequential data, rely on capturing long-term dependencies within time series. Noise can introduce errors in the early stages of the sequence, which may be continuously transmitted and accumulated through the recurrent connections in LSTM units, leading to incorrect learning of long-term dependent features. Consequently, LSTMs perform poorly on these datasets, with accuracies around 50%.

**Figure 11:**
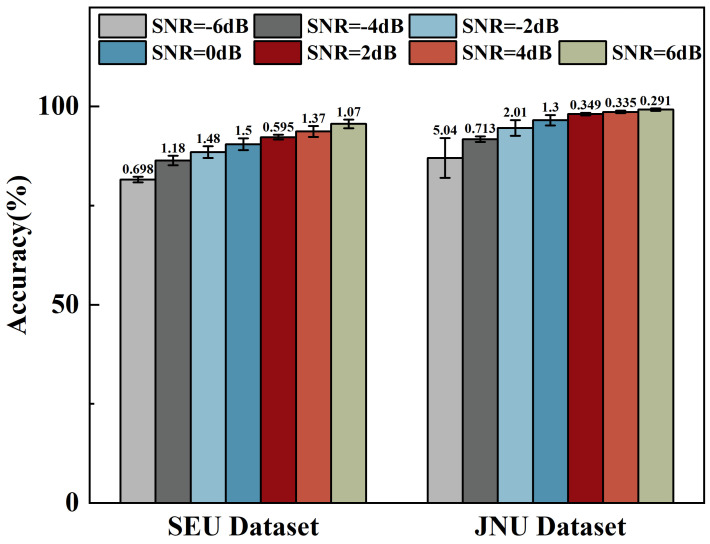
Rolling bearing fault diagnosis accuracies of KAN-HyperMP at seven noise levels.

Observing the KAN-HyperMP model, which aggregates information from higher-order neighbors, provides the model with a broader perspective. It relies not only on direct neighbors but can also gather features from a larger range of nodes. This extended view helps capture more complex and deep graph structural patterns. For instance, even if a node’s immediate neighbors are heavily affected by noise, introducing more distant neighbor nodes can dilute the noise’s impact with more effective information. Additionally, the model’s final part incorporates a B-spline-based KANLinear layer, which, due to its smoothness and local support characteristics, can handle and suppress input noise effectively. This helps to maintain the clarity of essential information at each network layer while filtering out unnecessary noise, as shown in Figure 11. Table 2 and Table 3 show that models using the KANLinear layer perform at 81.56% and 87.04% on the two datasets (SNR = −6 dB), respectively.

**Figure 12:**
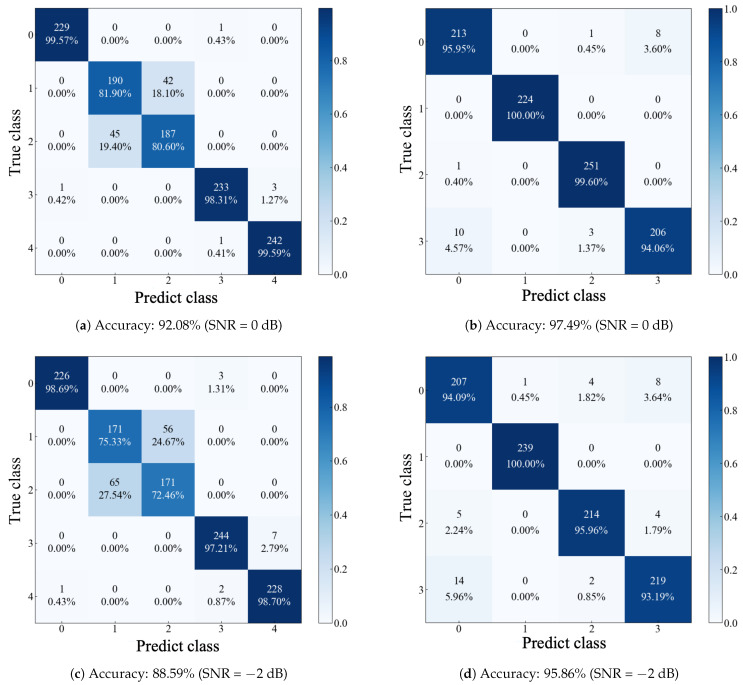

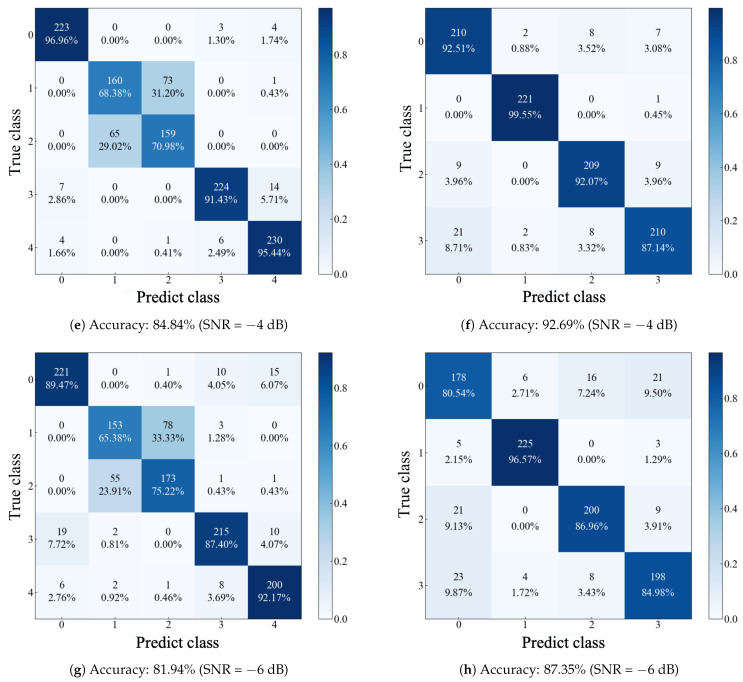
The confusion matrix of the proposed method. (1) Results (**a**,**c**,**e**,**g**) on the SEU Dataset; (2) Results (**b**,**d**,**f**,**h**) on the JNU Dataset.

Simultaneously, we used confusion matrices to visualize the results of the third experiment on two datasets (with SNR ranging from −6 dB to 0 dB). As illustrated in Figure 12a–h, the model’s performance on the JNU Dataset is noticeably superior to that on the SEU Dataset as the noise level increases. Specifically, within the SEU Dataset, the primary classification errors predominantly involve samples labeled 1 and 2.

In summary, even under extreme noise conditions, KAN-HyperMP maintained higher accuracy compared to other models, highlighting its robustness and precision.

#### 3.3.3. Ablation Experiments

To investigate the impacts of the neighbor feature aggregation block, the feature fusion block, and the KANLinear block, ablation experiments are conducted across all datasets. Below is a concise overview of these variants:

1. **KAN-HyperMP-w/o HP**: Removing the Hadamard product from the neighbor feature aggregation block eliminated the capability for cross-node interaction.
2. **KAN-HyperMP-w/o FFB**: By omitting the feature fusion block, node features are merely added to high-order neighbor features without further integration.
3. **HyperMP-MLP**: This variant replaces the KANLinear block with a traditional MLP.

**Figure 13:**
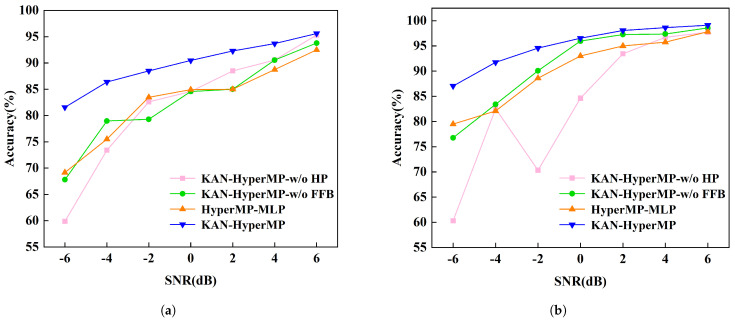
Fault-diagnosis accuracy of each block in the ablation experiments. (**a**) Experimental results on the SEU Dataset. (**b**) Experimental results on the JNU Dataset.

As shown in Figure 13a,b, removing the Hadamard product operation, the neighbor feature aggregation block lost its ability to facilitate cross-node interaction. This change occurred because the dot product operation, which performs element-wise multiplication on feature vectors of adjacent nodes, is eliminated. Normally, this operation not only merges features between nodes but also intensifies the non-linear relationships among them, capturing more complex dependencies. With its removal, the block can only combine feature vectors in a basic manner, lacking the intricate interactions needed. Consequently, this leads to reduced fault diagnosis accuracy, as noise interference in the data becomes more problematic without an effective feature interaction mechanism.

The feature fusion block enhances the model by merging node features with those of neighboring nodes, providing deeper insights into node interactions and introducing non-linear processing. This helps to capture the graph’s structure and node relationships from a broader, more “macro” perspective. However, without the feature fusion block, the model merely adds node features to high-order neighbor features in a simplistic manner, diminishing its ability to distinguish between noise and useful signals.

When the KANLinear block is replaced with a traditional MLP for feature extraction, the model loses the noise suppression and smoothing capabilities of the B-spline function. Such a change complicates the distinction between useful signals and noise in high-noise environments, leading to a gradual degradation in performance as the noise levels increase. In conclusion, the analysis demonstrates the effectiveness of the three components within the overall model.

#### 3.3.4. Hyperparameters Discussion

**Figure 14:**
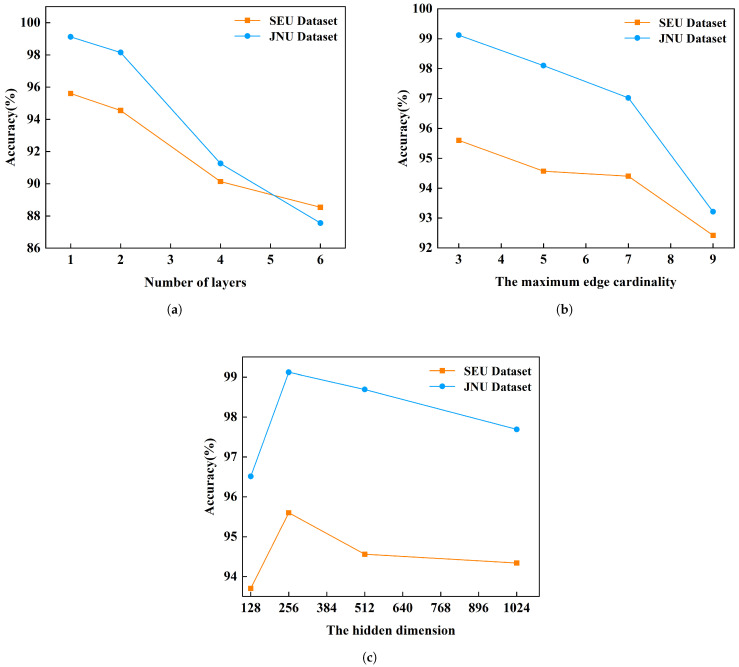
Parameter analysis on the classification performance of the proposed method. (**a**) The number of layers. (**b**) The maximum edge cardinality. (**c**) The hidden dimension.

Hyperparameter discussions are conducted under the condition of SNR = 6 dB, evaluating parameters such as the number of layers (neighbor feature aggregation block and feature fusion block), the hidden dimension of KAN-HyperMP, and the maximum edge cardinality, M. Tuning in noisy environments facilitates the identification of the optimal hyperparameters by striking a balance where the model minimizes noise interference without overfitting and losing its fault diagnosis capabilities in new data. The corresponding experimental results are depicted in Figure 14.

As shown in Figure 14a, model accuracy gradually decreases as the number of layers increases, with the optimal number being 1. At this stage, the fault diagnosis accuracy for the SEU and JNU datasets reaches 95.60% and 99.12%, respectively, though increasing the layers to 4 reduces accuracy to 88.54% and 87.56%. While adding layers is expected to deepen the model’s capacity to capture complex data features, in some hypergraph neural network architectures, aggregating information from more neighbors with each additional layer may dilute useful information, making node feature representations more similar and reducing the distinction between nodes, particularly when processing graph data.

Additionally, as depicted in Figure 14b, the model achieves its highest accuracy when M is set to 3. However, as M increases to 9, the accuracy decreases to 92.42% and 93.21% on the SEU and JNU datasets, respectively. This decline in performance with larger M values can be attributed to nodes aggregating features from more distant neighbors, which may have weaker relevance to the current node, thus introducing more noise into the data. Particularly in noisy environments, this information from distant neighbors may not only be unhelpful but could actually disrupt the correct interpretation of the current node’s state. Relative to the first two hyperparameters, variations in the hidden dimensions exert a less pronounced impact on accuracy. However, it is observed that the model attains its highest accuracy levels on the SEU and JNU datasets when the hidden dimensions are set to 256, as illustrated in Figure 14c.

## 4. Conclusions

In this paper, an innovative rolling bearing fault diagnosis method called KAN-HyperMP is developed. This method utilizes hypergraph theory to effectively identify and aggregate high-order neighbor node features. By applying B-spline functions within KAN, the smoothness of data processing is enhanced, thereby improving the accuracy of fault diagnosis and the stability of the model in noisy environments. Experimental results demonstrate that KAN-HyperMP exhibits exceptional fault detection capabilities and robustness, even under conditions of high noise, effectively addressing the challenges of complex fault diagnosis.

Although the proposed model has demonstrated commendable performance under extreme noise conditions, there is potential for further improvement in its accuracy. Consequently, future research will focus on enhancing the model’s robustness. Advanced noise filtering technologies and data augmentation strategies are planned to be incorporated to bolster performance in complex environments. Additionally, multimodal data fusion techniques will be explored to enrich the sources of information for fault diagnosis. These enhancements are expected to improve the model’s accuracy and applicability, better meeting the demands of industrial applications. Through these efforts, further optimization of the model is aimed to be achieved, ensuring its reliability in challenging conditions.

## Figures and Tables

**Table 1:** Model comparison using the SEU and JNU Datasets (without noise).

	SEU		JNU	
Model	Accuracy	F1-Score	Accuracy	F1-Score
CNN	98.60%	98.60%	99.02%	99.02%
LSTM	98.84%	98.80%	95.57%	94.80%
GCN	98.57%	98.53%	93.60%	93.70%
GAT	92.30%	92.70%	83.80%	83.80%
HGNN	98.99%	98.98%	90.40%	90.30%
HyperGCN	98.94%	98.93%	93.40%	93.20%
**KAN-HyperMP**	**99.70%**	**99.70%**	**99.10%**	**99.10%**

**Table 2:** Rolling bearing fault diagnosis on the SEU Dataset at seven noise levels.

Model	−6 dB	−4 dB	−2 dB	0 dB	2 dB	4 dB	6 dB
CNN	75.02%	79.40%	86.00%	93.20%	74.53%	72.69%	95.51%
LSTM	59.42%	57.05%	65.51%	70.05%	76.13%	83.06%	86.80%
GCN	60.10%	70.50%	76.00%	82.00%	82.70%	85.70%	87.30%
GAT	56.60%	66.60%	73.40%	72.90%	79.80%	81.60%	84.10%
HGNN	54.70%	64.80%	75.10%	78.10%	86.60%	80.00%	82.80%
HyperGCN	63.10%	67.00%	75.30%	81.90%	83.50%	89.00%	87.00%
**KAN-HyperMP**	**81.56%**	**86.37%**	**88.50%**	**90.47%**	**92.28%**	**93.69%**	**95.60%**

**Table 3:** Rolling bearing fault diagnosis on the JNU Dataset at seven noise levels.

Model	−6 dB	−4 dB	−2 dB	0 dB	2 dB	4 dB	6 dB
CNN	76.60%	85.71%	87.39%	94.00%	97.00%	98.40%	98.10%
LSTM	51.23%	64.23%	75.43%	81.36%	87.83%	81.12%	93.51%
GCN	56.20%	69.20%	74.10%	79.80%	85.80%	87.20%	88.50%
GAT	56.00%	65.60%	72.40%	77.90%	80.30%	80.40%	82.90%
HGNN	47.80%	62.10%	65.50%	70.90%	83.40%	80.20%	78.20%
HyperGCN	63.00%	66.00%	71.40%	71.90%	83.40%	85.27%	88.60%
**KAN-HyperMP**	**87.04%**	**91.76%**	**94.57%**	**96.54%**	**98.08%**	**98.64%**	**99.12%**

## Data Availability

The SEU and JNU Datasets provided in this study can be found in the following repository: https://github.com/Tan-Qiyu/Mechanical_Fault_Diagnosis_Dataset (accessed on 1 October 2024).
